# Transfusion-Related Acute Lung Injury in an Alcoholic Hepatic Cirrhosis Patient: A Case Report

**DOI:** 10.7759/cureus.35677

**Published:** 2023-03-02

**Authors:** Jonathan Livingston, Gowry Reddy

**Affiliations:** 1 Internal Medicine, New York Medical College at St. Mary’s General Hospital and St. Clare’s Health, Denville, USA

**Keywords:** hla antibody, transfusion, pulmonary edema, alcoholic cirrhosis, trali

## Abstract

Transfusion-related acute lung injury (TRALI) is one of the leading causes of transfusion-related mortality in the United States. It is a rare, potentially fatal complication of blood product transfusion, often seen in one in 5000 transfusion cases. On average, studies show a reported estimated fatality rate of 5-24% with a mortality rate of 12%. In the US, TRALI has been responsible for 30% of transfusion-related deaths. In this report, we discuss a case of a 51-year female with a past medical history of alcohol dependence and depression who presented complaining of dizziness and lightheadedness for 1 week. Subsequent diagnostic assessment and therapeutic interventions included various imaging studies, serial hematological evaluations, and eventual administration of blood transfusions, intravenous corticosteroids, supplemental oxygenation, and diuresis for clinical management. The occurrence of TRALI is often underreported due to a lack of timely recognition resulting in delayed treatment. Overall, we were able to not only diagnose TRALI in this patient but also effectively comprehend the significance of guiding appropriate management strategies due tohuman leukocyte antigen (HLA) TRALI-mediating antibodies to potentially reduce the overall incidence of such transfusion reactions.

## Introduction

Transfusion-related acute lung injury (TRALI) is one of the leading causes of transfusion-related mortality in the United States. It is a rare, potentially fatal complication of blood product transfusion, often seen in one in 5000 transfusion cases [1-3]. Understanding and appropriately differentiating TRALI from other diagnoses, such as transfusion-associated circulatory overload (TACO), acute febrile or nonfebrile hemolytic transfusion reactions, anaphylactic reactions, bacterial infections (HCAP), bronchiolitis organizing pneumonia (BOOP), pneumonitis, etc., can lead to timely therapeutic interventions. Early detection of disease paradoxically increases the incidence of diseases; however, it can prevent serious complications in treatable diseases.

## Case presentation

We present a case of a 51-year-old female with a past medical history of alcohol dependence and depression with chief complaints of lightheadedness for 1 week. Her pertinent social history was significant for unquantifiable alcohol consumption, with her last drink approximately 10 days ago.

At the time of admission, on physical examination, the patient was afebrile and normotensive, with oxygen saturation (SpO2) of 96% on ambient air. Pertinent physical exam findings include: bilateral scleral icterus and conjunctival pallor, jaundiced skin, and spider angiomata on the abdominal trunk and upper extremities. Abdominal ascites and lower extremities with 1+ pitting edema bilaterally were noted.

A hematologic investigation was performed, including a complete blood count, which revealed anemia (hemoglobin (Hb) 4.8 g/dl), macrocytosis (mean corpuscular volume 118 fl), and mild thrombocytopenia (platelet count 132/mcL). Pro-brain natriuretic peptide (pro-BNP) was 61.8. Laboratory studies: aspartate aminotransferase/alanine aminotransferase 177/84, total bilirubin 10.1, prothrombin time/international normalized ratio 9/0.93; ammonia level 38 (normal). Complete blood count: WBC 12.8 (mild leukocytosis), low Hb (4.8 g/dl), hematocrit of 13.9, platelets 132/mcL. Ultrasound abdomen showed evidence of liver cirrhosis, splenomegaly, and a small amount of ascites. CT abdomen and pelvis showed liver cirrhosis with ascites. An initial diagnosis of symptomatic macrocytic anemia in the setting of alcoholic cirrhosis was made.

On hospital day 1, the patient was transfused with three units of packed red blood cells due to a lack of significant improvement in Hb after the second unit; hemoglobin improved to 7.4 g/dl after the third unit. On hospital day 2, the patient’s hemoglobin further dropped to 6.6 g/dl, for which an additional one unit of packed red blood cells was transfused, for a total of four units during hospitalization. Shortly after the last unit was transfused, the patient complained of shortness of breath, and her oxygen saturation reduced.

**Figure 1:**
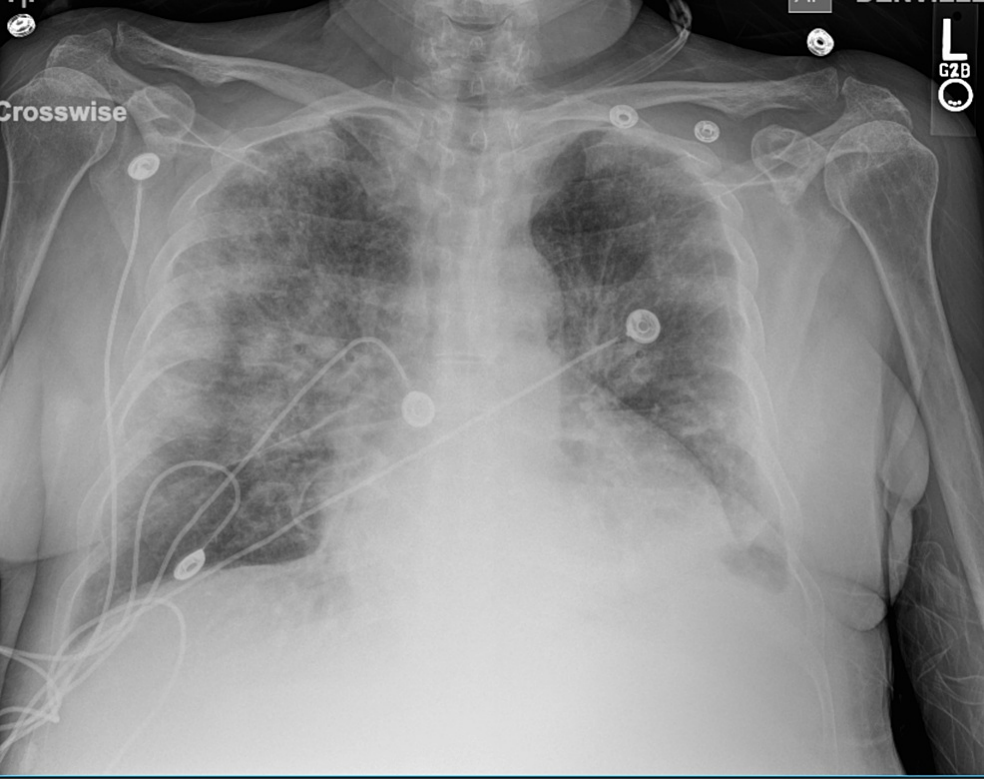
Chest X-ray showing bilateral pulmonary edema with bilateral patchy infiltrates suggestive of TRALI TRALI: transfusion-related acute lung injury

Pertinent physical exam findings at the time of the last transfusion included vital signs: blood pressure 90/60 mmHg, heart rate 70 breaths/min, SpO2 82%, and diminished breath sounds to auscultation bilaterally. Additional work-up included a chest X-ray which showed bilateral pulmonary infiltrates (Figure 1) and a pro-BNP low-normal of ~62. Consequently, the patient received IV methylprednisolone q6h for a course of three days; the patient was noted to be improving on day 2 of the administration of IV steroids. She had a one-time febrile episode which was treated with empiric IV piperacillin-tazobactam and later de-escalated.

The patient was placed on a nasal cannula for supplemental oxygen therapy for the course of four days. She later improved by hospital day 6 and was discharged to a rehabilitation facility on a prednisone taper regimen.

Various differential diagnoses of TRALI include transfusion-associated circulatory overload (TACO), acute febrile or nonfebrile hemolytic transfusion reactions, anaphylactic reactions, and bacterial respiratory infections (healthcare-associated pneumonia, community-acquired pneumonia, bronchiolitis obliterans organizing pneumonia [BOOP], and pneumonitis). In this case, most of the clinical findings assimilated to TRALI as depicted in Table 1 [4].

**Table 1:** TRALI versus TACO TRALI: transfusion-related acute lung injury, TACO: transfusion-associated cardiac overload, CXR: chest X-ray, BNP: B type natriuretic peptide, PAOP: pulmonary artery occluding pressure. Source: Kim et al. [4]

	TRALI	TACO
Blood pressure	Low-normal	Normal-high
Body temperature	Normal-elevated	Normal
CXR	No vascular congestion	Vascular congestion, pleural effusion
BNP	Low (< 250 pg/ml)	High
PAOP	Low-normal	High
Ejection fraction	Normal function	Abnormal function
Response to diuretics	Inconsistent	Improved
Edema fluid	Transudate	Exudate

Long-term follow-up portrayed two courses of hospitalization requiring the patient to receive blood transfusions; however, she subsequently passed away due to uncontrolled refractory esophagovariceal bleeding a year later. During these hospitalizations, she did not develop recurrent episodes of TRALI.

## Discussion

TRALI has been defined as new-onset acute lung injury occurring within hours of blood transfusion. Some common precipitating risk factors include acute infections, burns, shock, sepsis, coagulopathy, massive transfusion, pneumonia, chronic alcoholism, leading to end-stage liver disease (ESLD), acute renal failure, malignancy undergoing chemotherapy with absolute neutrophil count (ANC)>500, pneumonia, cardiopulmonary bypass surgery, etc. [5-6]. 

This case meets the diagnostic criteria for TRALI, specifically Type 1, during and within 6 hours of transfusion; its associated clinical findings of extensive bilateral infiltrates on chest radiograph, with no evidence of pulmonary vascular overload, and hypoxemia (SpO2<90%) are delineated in Table 2 [7]. Of note, diagnosis of TRALI is primarily a clinical diagnosis and does not necessarily depend on serological investigation; nonetheless, some studies describe that a typical TRALI serologic workup consists of testing human leukocyte antigen (HLA) Class I and II and neutrophil-specific antibodies. Additionally, histological findings demonstrate pulmonary edema, capillary leukostasis, and neutrophil extravasation [8-13]. To further elaborate, the pathophysiology of TRALI is largely extensive and often remains overlooked; the two-hit neutrophil activation hypothesis is often described in the literature, where the result of the first hit involves the clinical condition of the patient and the second hit involves the blood transfusion product. Specifically, in TRALI, the first hit tends to be inflammation, subsequently followed by a second hit triggered by anti-leukocyte antibodies or biological response modifiers, such as lipids [12].

In our case, the first hit was largely defined by the patient’s underlying risk factor of ESLD. As described in one specific study by Benson et al., transfused patients with ESLD (n = 72) developed TRALI more frequently than those without ESLD (29% versus 1%, p < 0.01) [14]. Various pathways have been described in multiple studies, such as those that are monocyte and neutrophil dependent, complement and monocyte dependent, and neutrophil Fc receptor, platelet and neutrophil extracellular trap dependent. Other pathways mitigated by direct endothelial cell activation and direct monocyte activation have also been described [10-13]**. **

The severity of TRALI can depend on targeted antigen-antibody interactions; particularly caused by antibodies against the human neutrophil antigen (HNA-3a) involved in severe and fatal TRALI cases [15]. Administration of plasma-rich blood components from male donors or donors with negative leukocyte antibody screening tests as a treatment strategy can effectively decrease the severity of TRALI cases [15-17]. Serological investigations, such as leukocyte antibody screening tests and cytokine measurements, can guide to prognosticate the severity of TRALI mitigated by these antibody-driven processes. In our case, the clinical decision-making process entailed clinical diagnosis and treatment with serial radioimaging to monitor the progression of symptom resolution. Hence, understanding these underlying mechanisms precipitating TRALI can allow us to better delineate treatment strategies in addition to clinical recognition of symptoms. 

## Conclusions

This case illustrates the potential for transfusion-related acute lung injury secondary to multiple packed red blood cell transfusions. It is crucial to acknowledge pertinent risk factors, such as ESLD, in addition to identification of clinical signs of TRALI for establishing a proper diagnosis. Recognition of this clinical syndrome is not only important in guiding appropriate management strategies, but it is also imperative in prompting further investigation of blood donors, which can more specifically identify potential life-threatening HLA TRALI-mediating antibodies, thus leading to a possible reduction in the incidence of one of the principal causes of mortality in transfusion reactions.

## Appendices

**Table 2:** Diagnostic Criteria of TRALI Subtypes TRALI: transfusion-related acute lung injury; ARDS: acute respiratory distress syndrome Source: Vlaar et al. [7]

TRALI Type I	TRALI Type II
No risk factors for ARDS and following criteria met: Acute onset (within 6 hours) Hypoxemia Bilateral pulmonary edema on X-ray No evidence of left arterial hypertension/left circulatory overload Onset during or within 6 hours of blood transfusion No temporal relationship to alternative risk factor for ARDS (no pre-existing acute lung injury/ARDS before transfusion)	Risk factors for ARDS present (no overt diagnosis of ARDS) or mild ARDS at baseline with respiratory compromise possibly due to transfusion based on following criteria Findings as described in TRALI type I Stable respiratory status 12 hours before transfusion
